# Independent Risk Factors and a New Nomogram for Predicting Breast Cancer Risk for Bone Metastasis in Chinese Women: A Retrospective Study with External Validation

**DOI:** 10.3390/jcm15062324

**Published:** 2026-03-18

**Authors:** Yunfei Huang, Tianjiao Ge, Heng Song, Wenjia Zhang, Meiqi Wang, Zhenchuan Song

**Affiliations:** Breast Center, The Fourth Hospital of Hebei Medical University, Shijiazhuang 050017, China; hyf150310818861998@163.com (Y.H.); gtianj@yeah.net (T.G.); songh0104@yeah.net (H.S.); zwj17856276918@163.com (W.Z.); maggie92320@hotmail.com (M.W.)

**Keywords:** breast cancer, bone metastases, nomogram, seer database, predictive model

## Abstract

**Background/Objectives:** Bone is the most common organ affected by distant metastasis in advanced breast cancer, and the development of skeletal-related events (SREs) often leads to significant deterioration in patients’ quality of life and survival outcomes. In this study, we aimed to explore the risk factors associated with bone metastasis in breast cancer and to develop a predictive nomogram for identifying high-risk patients, which may facilitate timely preventive interventions and improve clinical prognosis. **Methods:** A retrospective analysis was conducted on 672 patients with breast cancer who underwent surgery at the Fourth Hospital of Hebei Medical University (Shijiazhuang, China) between 2013 and 2023; this cohort served as the training set. Clinical and pathological characteristics potentially influencing bone metastasis—including age, menopausal status, histological grade, affected side, maximum tumor diameter, lymph node staging, TNM staging, ER status, PR status, HER-2 status, Ki-67, molecular subtypes, vascular tumor thrombus, nerve infiltration and visceral metastasis—were collected. The median follow-up time was 42 months. Patients were stratified into two cohorts based on whether postoperative bone metastasis occurred, with groups matched according to Tumor–Node–Metastasis (TNM) stage. Univariate and multivariate logistic regression models were applied to identify independent factors associated with breast cancer bone metastasis, and a nomogram prediction model was constructed using the variables retained in the final analysis. For external validation, data from 2814 patients with breast cancer who underwent surgery between 2013 and 2021 were extracted from the U.S. Surveillance, Epidemiology, and End Results database. **Results:** The multivariate logistic regression analysis revealed that histological grade (*p* = 0.002), progesterone receptor (PR) negativity (*p* = 0.001), human epidermal growth factor receptor 2 (HER-2) negativity (*p* = 0.002) and visceral metastasis (*p* < 0.001) were identified as independent predictors of bone metastasis in breast cancer. A nomogram predictive model was established using these four factors. The area under the receiver operating characteristic curve was 0.720 (95% confidence interval (CI): 0.6797–0.7607) for the training cohort and 0.701 (95% CI: 0.6813–0.7205) for the external validation cohort. Decision curve analysis further confirmed the clinical applicability of the model. **Conclusions:** The present study confirms that histological grade, PR status, HER-2 status and visceral metastasis are independent factors associated with bone metastasis in breast cancer. The constructed nomogram may effectively predict breast cancer-related bone metastasis and could serve as a practical tool for clinical decision-making.

## 1. Introduction

Breast cancer is the most commonly diagnosed malignancy among women worldwide. According to the Global Cancer Observatory (GLOBOCAN 2022), approximately 2.3 million new breast cancer cases were diagnosed globally in 2022, accounting for nearly 24% of all cancers in women. posing a severe threat to both physical and mental health [1,2]. Bone represents the most frequent site of distant metastasis in patients with breast cancer. Clinical studies have shown that nearly 65–75% of individuals with advanced breast cancer ultimately experience the development of skeletal metastases during disease progression. The occurrence of bone metastasis is commonly accompanied by a variety of skeletal-related events (SREs), such as pathological fractures, spinal cord compression, hypercalcemia, and intense bone pain. These complications can markedly deteriorate patients’ quality of life and are often associated with prolonged hospitalization, increased medical expenditures, and poorer survival outcomes [3]. Despite notable advancements in systemic therapy for breast cancer, advanced-stage disease remains incurable [4]. Chinese patients with breast cancer present distinct epidemiological and biological characteristics, which creates a clinical gap compared with Western populations, and necessitates the development of population-specific diagnostic and therapeutic tools [5]. Notably, the proportion of bone metastasis combined with other metastatic sites at first recurrence is higher among Chinese patients with breast cancer than in patients from developed countries. For example, a German cohort study reported that bone metastasis accounted for 80.7% of first recurrences, with isolated bone metastasis comprising 35.3% [6]. By contrast, a Chinese retrospective study (CSBrS-023) revealed that bone metastasis constituted 87.7% of first recurrences, whereas isolated bone metastasis only accounted for 26.5% [7].

Identifying patients at risk of bone metastasis and predicting their survival outcomes is essential to guide subsequent clinical examinations, treatments and follow-up management. Distant metastasis-free survival in breast cancer may be influenced by various clinicopathological factors, including lymphovascular invasion (LVI), Ki-67 expression, human epidermal growth factor receptor 2 (HER-2)status, estrogen receptor (ER)/progesterone receptor (PR) status, lymph node staging and tumor size [8]. However, the contribution of biological factors to bone metastasis in breast cancer remains incompletely understood, and published findings have shown considerable variability [9]. Further studies are warranted to identify effective predictors of bone metastasis risk.

Nomograms are reliable and user-friendly prognostic tools that integrate quantitative risk factor analysis, making them widely applicable in various cancer types [10,11]. Although multiple tools exist to assist in breast cancer diagnosis and treatment decisions, most are developed based on data from Western populations [9,12,13]. Biomarkers, genetics, lifestyle and socioeconomic status exert varying effects on breast cancer across different ethnic groups [14,15,16]. Consequently, it is imperative to develop a predictive model for assessing the risk of bone metastasis in breast cancer that is specifically based on clinical data from Chinese patients. In this study, we retrospectively collected and analyzed the clinicopathological data of breast cancer patients treated at a tertiary medical center in China. By integrating clinicopathological characteristics with molecular markers, we aimed to develop and validate a risk prediction model for bone metastasis in Chinese patients with breast cancer. The model was further externally validated using data from the U.S. Surveillance, Epidemiology, and End Results (SEER) database. Specifically, this study sought to identify independent risk factors associated with bone metastasis and to construct a predictive nomogram incorporating clinicopathological variables and molecular markers to identify high-risk patients. Such a model may facilitate early preventive interventions and potentially improve clinical outcomes.

## 2. Materials and Methods

### 2.1. Patient Population

The current study retrospectively analyzed clinical data from female patients with breast cancer who underwent surgery at the Fourth Hospital of Hebei Medical University (Shijiazhuang, China) between January 2013 and December 2023. Inclusion criteria: (1) 18 years of age or older; (2) Histopathologically confirmed primary breast cancer; (3) Female patients who underwent surgical treatment; (4) Availability of complete clinicopathological and immunohistochemical data. Exclusion criteria: (1) Presence of distant metastasis at the time of initial diagnosis; (2) Incomplete clinical or pathological data; (3) Male breast cancer. The diagnosis of breast cancer was pathologically confirmed and bone metastasis was diagnosed based on imaging evidence from bone scintigraphy, computed tomography (CT), magnetic resonance imaging (MRI), or positron emission tomography–computed tomography (PET-CT). Patients were divided into two groups: A postoperative bone metastasis recurrence group and a postoperative non-bone metastasis recurrence group, matched by Tumor-Node-Metastasis (TNM) staging.

A total of 672 breast cancer patients who satisfied the predefined inclusion criteria were enrolled in the training cohort and subsequently subjected to univariate and multivariate logistic regression analyses. For external validation, clinical data from 2814 female breast cancer patients who underwent surgical treatment between 2013 and 2021 were obtained from the U.S. Surveillance, Epidemiology, and End Results (SEER) database (https://seer.cancer.gov/data/access.html) (accessed on 22 November 2024).

### 2.2. Data Collection

The following clinicopathological variables were retrieved from the patients’ medical records: Age (the median age at diagnosis was 51 years (range, 23–80 years)), menopausal status, histological grade, affected side, maximum tumor diameter, lymph node staging, TNM staging, ER status, PR status, HER-2 status, Ki-67, molecular subtypes, vascular tumor thrombus, nerve infiltration and visceral metastasis. Pathological specimens were collected from the patients and subsequently subjected to immunohistochemical examination. All procedures were conducted in the ancillary departments of our institution. To assess the role of visceral metastasis as a potential risk factor for subsequent bone metastasis, only patients whose visceral metastasis was confirmed prior to the detection of bone metastasis or patients for whom both metastases were detected simultaneously were recorded and included in the bone metastasis-positive group. This ensured that visceral metastasis function was assessed as a time-ordered predictive risk factor rather than merely a disease severity marker. Risk stratification of patients with cancer was performed using the current TNM staging system (versions 6.0 and 7.0) of the American Joint Committee on Cancer (AJCC) [17].

### 2.3. Diagnostic Criteria

Immunohistochemistry (IHC) was applied to evaluate the expression of ER, PR, HER-2, and Ki-67. Based on the 2020 recommendations issued jointly by the American Society of Clinical Oncology and the College of American Pathologists (2020 ASCO/CAP), ER or PR positivity was defined as nuclear staining in at least 1% of tumor cells, while staining observed in fewer than 1% of nuclei was regarded as negative [18]. HER-2 expression was evaluated using immunohistochemistry (IHC). An IHC score of 0 or 1+ indicated HER-2 negativity, while a score of 3+ was interpreted as positive. Samples with an equivocal score of 2+ underwent additional fluorescence in situ hybridization (FISH) testing to determine HER-2 gene amplification. Cases lacking HER-2 amplification were classified as negative, whereas amplification confirmed by FISH was defined as positive. [19,20]. The Ki-67 index was calculated as the percentage of nuclear-stained tumor cells in the hotspot area. The optimal cutoff value for Ki-67 remains a subject of ongoing debate. Based on the 2021 St. Gallen Breast Cancer Guidelines and our institutional clinical practice, a Ki-67 index ≥ 30% was defined as high expression [21].

### 2.4. Statistical Analysis

All statistical analyses were performed using IBM SPSS Statistics 22.0 (IBM). Continuous data are expressed as the mean ± standard deviation, while categorical variables are reported as percentages or proportions. Comparisons of categorical variables between groups were conducted using the χ^2^ test or Fisher’s exact test. The training cohort was subjected to univariate analysis to screen for possible risk factors contributing to bone metastasis. According to the 2021 St. Gallen Breast Cancer Guidelines, molecular subtypes were highly correlated with ER status, PR status, and (HER-2) status. Additionally, the excessive number of molecular subtype groups is not conducive to model construction. After discussion by the research team, molecular subtypes were not included for further research [21]. Factors identified as significant were further entered into multivariate logistic regression analysis. The outcomes were expressed as odds ratios (ORs) along with their 95% confidence intervals (95% CIs). All statistical tests were two-tailed, and *p* < 0.05 was regarded as statistically significant.

A bone metastasis risk predictive model was constructed using R software version 4.2.2 (http://www.r-project.org) (accessed on 11 December 2024) and MSTATA software version 0.93 (www.mstata.com) (accessed on 11 December 2024). Univariate and multivariate logistic regression analyses were conducted to identify independent risk factors associated with bone metastasis in breast cancer. Variables with *p* < 0.05 in univariate analysis were included in the multivariate logistic regression analysis. The backward stepwise regression method was employed for independent risk factor screening. A nomogram model was established based on the results of univariate and multivariate logistic regression analyses and was validated through an external validation cohort. To evaluate the predictive accuracy of the nomogram, receiver operating characteristic (ROC) curves were generated and the area under the curve (AUC) was calculated. An AUC value exceeding 0.7 indicated that the nomogram had reasonable predictive value. The performance of the model was assessed using calibration curves and decision curve analysis (DCA).

## 3. Results

### 3.1. Baseline Characteristics of the Training and Validation Cohorts

The baseline demographic and clinical characteristics of the training and validation cohorts were evaluated. A total of 3486 patients were enrolled in this retrospective analysis. The training cohort (*n* = 672) from our center consisted of 224 patients with bone metastasis and 448 without. The external validation cohort from the SEER database (*n* = 2814) included 1609 patients with bone metastasis and 1205 without.

In the included training cohort and external validation cohort, the bone metastasis-positive patients were significantly older in the validation cohort (71.3% were >50 years old). In addition, the proportion of bone metastasis-positive patients with histopathological grade III cancer in the validation group was significantly higher (52%), and the hormone receptor (HR)+/HER-2- subtype was the main subtype in both groups (59.4% positive in the training cohort and 67.3% positive in the validation cohort). The detailed descriptive characteristics of the population are shown in Table 1.

### 3.2. Risk Factor Analysis

Univariate and multivariate analyses were conducted based on clinical pathological information to explore potential predictive factors for bone metastasis in the training cohort. As shown in Table 2, in the univariate analysis, histological grade, ER, PR, HER-2, Molecular subtype, KI67 and visceral metastasis were all significantly different (all *p* < 0.05). Molecular subtype was not incorporated into the model because it is strongly associated with ER, PR, and HER-2 status. In addition, the inclusion of multiple molecular subtype categories may increase model complexity. Furthermore, Ki-67 was not included in the subsequent analysis because this variable is not recorded in the Surveillance, Epidemiology, and End Results (SEER) database. After discussion among the research team, molecular subtype and Ki-67 were therefore excluded from further analyses. After incorporating these five variables into logistic regression, the model fit well (Hosmer–Lemeshow test χ^2^ = 5.170, *p* = 0.396), and histological grade [OR = 0.516, 95% CI (0.337–0.790)], PR status [OR = 0.476, 95% CI (0.308–0.736)], HER-2 status [OR = 0.502, 95% CI (0.327–0.771)] and visceral metastasis [OR = 26.831, 95% CI (12.230, 58.864)] were all factors affecting bone metastasis.

### 3.3. Performance of the Nomogram

**Figure 1:**
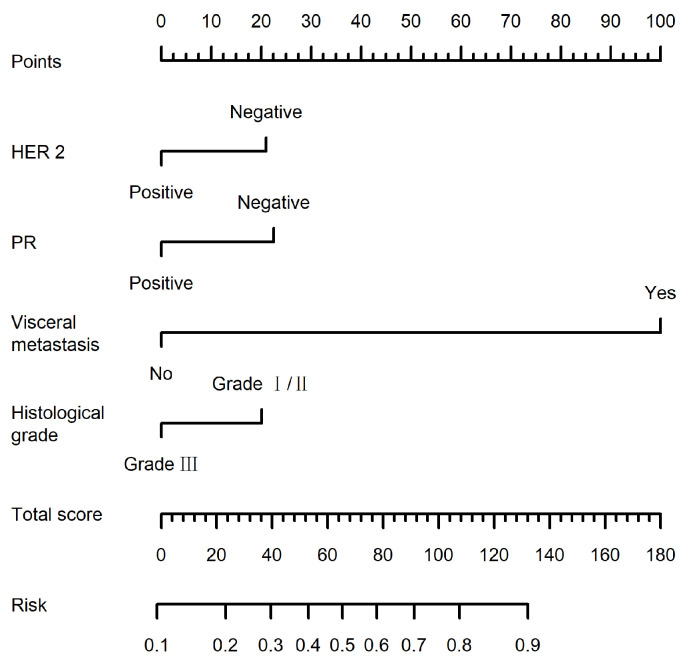
Nomogram predicting the probability of bone metastasis.

A predictive model was developed based on the four independent risk factors identified and visualized as a nomogram (Figure 1). For each patient, a score was assigned to each of the four predictors; the total score was used to estimate the probability of bone metastasis. As shown in Figure 1, visceral metastasis had the highest weight (100 points), followed by PR negativity (22.6 points), indicating that patients with visceral metastasis are more likely to develop bone metastasis. Factors associated with an increased risk of bone metastasis included histological grades I/II, PR negativity, HER-2 negativity and visceral metastasis.

**Figure 2:**
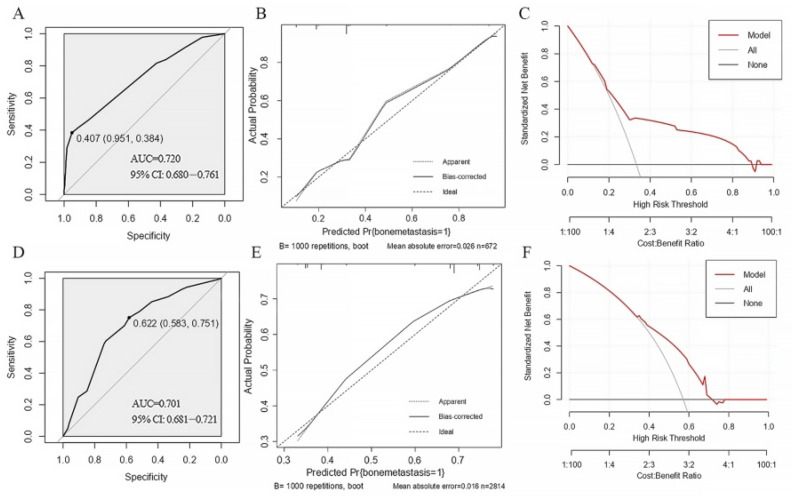
The ROC curves for the nomogram model are shown in (**A**) the training cohort (AUC = 0.720) and (**D**) the validation cohort (AUC = 0.701). Calibration curves are plotted for the (**B**) training cohort and (**E**) validation cohort. The “Ideal” line represents perfect prediction accuracy. The “Apparent” line indicates agreement between predicted and observed probabilities. The “Bias-corrected” line shows the adjusted prediction accuracy. Better agreement between predicted and observed values is indicated when the apparent or bias-corrected curve lies closer to the ideal line. Decision curve analysis (DCA) for the nomogram model in the (**C**) training cohort and (**F**) validation cohort. The y-axis represents net benefit. The “Treat All” line assumes all patients have postoperative bone metastasis recurrence, while the “Treat None” line assumes no patients experience recurrence. The solid line represents the nomogram’s net benefit when guiding clinical decisions across different threshold probabilities (x-axis). AUC: area under the ROC curve; CI: confidence interval.

The AUC value of the nomogram was 0.720 (95% CI: 0.6797–0.7607) for the training cohort (Figure 2A) and 0.701 (95% CI: 0.6813–0.7205) for the validation cohort (Figure 2D). Furthermore, a 1000-time bootstrap resampling method was used to verify model calibration: The predicted probabilities were closely aligned with the actual observed probabilities in both the training cohort (Figure 2B) and validation cohort (Figure 2E), confirming good predictive accuracy.

DCA was used to evaluate the clinical utility of the model. In the training cohort, when the threshold probability of bone metastasis was between 20 and 90%, the net benefit of using the nomogram to guide clinical decisions was higher than that of either treating all patients or treating none (Figure 2C). In the validation cohort, the nomogram provided greater net benefit than the two extreme strategies when the threshold probability was between 30 and 70% (Figure 2F).

## 4. Discussion

The present study successfully constructed and validated a bone metastasis risk prediction model for Chinese patients with breast cancer by integrating clinicopathological characteristics and molecular markers. The results not only identified independent risk factors for bone metastasis but also developed a clinically practical tool with good discriminative ability, providing a basis for personalized treatment decisions.

While several risk prediction models have been developed for breast cancer, the included population with breast cancer is typically small (200 cases) compared with the SEER database, affecting the precision of the model [1]. Moreover, prediction models (e.g., the AJCC-TNM staging system) derived from Western cohorts demonstrated limited predictive performance in Chinese patients, probably due to the differences in biological characteristics between Chinese and Western populations [17,22,23,24,25]. Validation in both the training and validation cohorts demonstrated good performance of the nomogram, with AUC values of 0.720 and 0.701, respectively, supporting the robustness of the model derived from a large population of Chinese patients. Through multivariate analysis, four independent risk factors for breast cancer bone metastasis were identified. Consistent with previous studies [23,25], tumor grade and molecular subtype were confirmed as risk factors for metastatic breast cancer. Specifically, the current study verified that histological grade, PR status, HER-2 status and visceral metastasis were independent predictors of bone metastasis. The strong predictive power of visceral metastasis on subsequent bone metastasis (OR = 26.831) warrants careful interpretation, because it introduces predictor overlap and risk of circular inference. Notably, both visceral metastasis and bone metastasis are manifestations of systemic dissemination. To address this concern, visceral metastasis in the bone metastasis-positive group was strictly defined as an event identified either prior to or simultaneously with the occurrence of bone metastasis during follow-up. Such a temporal sequence would support its role as a predictive factor rather than merely a co-outcome. Visceral metastasis may biologically alter systemic physiology or release cytokines and exosomes that prime the bone marrow microenvironment, further facilitating subsequent skeletal colonization. This finding also aligns with the clinical situation that the proportion of patients with breast cancer with concurrent visceral and bone metastases at first recurrence is markedly higher in China than in Western countries. The high OR value of visceral metastasis in the present study supports the findings of Liang et al. [4,5,26]; these previous studies proposed that visceral metastasis should be regarded as a ‘signaling node’ for systemic disease management rather than an isolated lesion. Timely intervention to block the promotional effect of visceral metastasis on bone metastasis may improve overall prognosis.

In terms of clinical application, the nomogram provides a practical tool for risk stratification and may be suitably applied during the postoperative follow-up for patients without distant metastasis at initial diagnosis. Based on the calculated risk, patients can be stratified into distinct pathways. Intensive surveillance is warranted for high-risk individuals, such as bone imaging (bone scintigraphy or PET-CT) every 6–12 months, even in the absence of symptoms. Additionally, physicians could consider initiating bone-targeted agents as primary prevention for patients at the highest risk, in accordance with emerging clinical guidelines and shared decision-making. Standard follow-up intervals should be maintained for low-to-moderate-risk patients to optimize resource allocation. However, the immediate clinical imperative remains comprehensive staging and systemic therapy for patients who have developed visceral metastasis; in this case, the model serves not for initial prediction but could help assess the possibility of subsequent skeletal involvement. Thus, the nomogram provides a structured framework to guide surveillance intensity and preventive strategies in the pre-metastatic setting.

In the current study, patients with HER-2-negative breast cancer were more likely to develop bone metastasis, which is consistent with the findings reported by Gong et al. (2018), who observed a similar trend in their cohort of breast cancer patients with bone metastases at initial diagnosis [27]. The HER-2 signaling pathway regulates osteoclast activity through the receptor activator of NF-κB ligand (RANKL)/osteoprotegerin (OPG) system. In HER-2-negative tumors, elevated levels of RANKL stimulate osteoclast activation, creating a “vicious cycle” that facilitates bone metastasis. In contrast, patients with HR-negative/HER-2-positive breast cancer and triple-negative breast cancer (TNBC) tend to develop visceral metastases more frequently. This could explain why these patients often succumb to visceral complications before bone metastasis is detected [28,29,30,31,32].

PR-negative breast cancer may have a greater tendency to develop bone metastasis. This may be partly explained by the lack of progesterone-mediated protective signaling, which can disturb the physiological equilibrium between osteoblasts and osteoclasts within the bone microenvironment. Moreover, compared with PR-positive tumors, PR-negative breast cancers often display enhanced invasive and metastatic capabilities and may produce osteolytic factors, including parathyroid hormone-related protein (PTHrP). These factors can facilitate osteoclast formation by increasing receptor activator of NF-κB ligand (RANKL) expression in osteoblasts or stromal cells [9,33,34,35].

Histological grade was also identified as an independent prognostic factor for bone metastasis. Gong et al. [27] confirmed that tumor grade is an important predictor in various cancer prognostic models. Consistent with the present findings, a previous analysis of the SEER database has shown that higher histological grades are associated with a lower risk of bone metastasis [9]. Notably, the current study observed that patients with lower histological grades (I/II) had a higher risk of bone metastasis, which contradicts conventional understanding. This may be attributed to the following explanations. Firstly, the factors affecting bone metastasis include not only histological grade, but also other unmeasured clinicopathological or biological factors. Despite their relatively low aggressiveness, lower-grade tumors are often enriched in luminal subtypes and promote bone metastasis due to specific molecular interactions, such as the CXCR4/CXCL12 axis [36,37]. Secondly, indolent and HR-positive tumors can exhibit a prolonged dormancy period, leading to late-onset metastasis that is disproportionately detected during follow-up [38,39]. Thirdly, cohort- and population-specific differences must be considered. Selection biases in cohort studies, such as the inclusion of more advanced symptomatic cases and the unique distribution of tumor microenvironment characteristics in Chinese patients, can markedly influence the observed association between grade and metastasis site [40]. Notably, the association identified in the present study should be regarded as an exploratory finding rather than a challenge to the established prognostic value of histological grades. Future studies should validate and elucidate this relationship through stratified analyses by molecular subtype (for example, interactions between histological grades and HR status), integration of multi-omics profiling and longitudinal data.

Furthermore, limitations remain in the present study. Firstly, the retrospective design of this study represents a limitation, and validation through larger prospective studies is still required. Secondly, the external cohort was derived from the SEER database, which lacked basic patient data, lifestyle information, genetic testing results and clinicopathological variable data (such as Ki-67, LVI and perineural infiltration). Consequently, the validation was partial, as it could not assess the predictive contribution of these unavailable factors. Moreover, notable population heterogeneity exists between the SEER cohort and the Chinese training cohort, with differences in biological characteristics, disease patterns and treatment standards. These differences could affect model calibration when applied to distinct populations, a phenomenon known as calibration drift. Therefore, the findings should be interpreted with an understanding of these inherent data and population limitations, while independent database validation supports the general framework of the model. Future multi-center prospective studies are needed to incorporate more comprehensive risk variables to enhance the accuracy and representativeness of the model for the target population. Thirdly, the use of logistic regression for bone metastasis is a methodological constraint in the retrospective cohort without accounting for time-to-event information, potentially causing biased estimates. However, this approach is considered pragmatic given the primary goal of identifying predictors within the observational period and the variability during follow-up, as the sequence of bone metastasis and visceral metastasis was described in the methodology.

## 5. Conclusions

In the present study, histological grade, PR status, HER-2 status, and visceral metastasis were determined to be independent factors related to the development of bone metastasis in breast cancer. Notably, a nomogram constructed using these factors demonstrated good predictive performance. Clinicians may use this nomogram to screen high-risk patients for bone metastasis, enabling timely detection and intervention during follow-up and treatment. This approach may help control tumor progression, prolong patient survival and delay the development of SREs.

## Figures and Tables

**Table 1:** Clinical and pathological characteristics of patients in the training and external validation cohorts. The characteristics include age, histological grade, affected side, Maximum tumor diameter, N stage, TNM stage, ER, PR, HER-2, Molecular subtype, and visceral metastasis.

Characteristics	Training Cohort		Validation Cohort	
	Bone metastasis (+)	Bone metastasis (−)	Bone metastasis (+)	Bone metastasis (−)
Cases	*n* = 224	*n* = 448	*n* = 1609	*n* = 1205
Age				
≤50	111 (49.6%)	211 (47.1%)	462 (28.7%)	340 (28.2%)
>50	113 (50.4%)	237 (52.9%)	1147 (71.3%)	865 (71.8%)
Histological grade				
I & II	164 (73.2%)	284 (63.4%)	772 (48.0%)	399 (33.1%)
III	60 (26.8%)	164 (36.6%)	837 (52.0%)	806 (66.9%)
Affected side				
left	118 (52.7%)	245 (54.7%)	812 (50.5%)	585 (48.5%)
right	106 (47.3%)	203 (45.3%)	797 (49.5%)	620 (51.5%)
Maximum tumor diameter				
≤2 cm	79 (35.3%)	184 (41.1%)	222 (13.8%)	166 (13.8%)
>2 cm ≤ 5 cm	138 (61.6%)	254 (56.7%)	778 (48.4%)	547 (45.4%)
>5 cm	7 (3.12%)	10 (2.23%)	609 (37.8%)	492 (40.8%)
N stage				
Positive	155 (69.2%)	296 (66.1%)	1358 (84.4%)	983 (81.6%)
Negative	69 (30.8%)	152 (33.9%)	251 (15.6%)	222 (18.4%)
ER				
Positive	172 (77.1%)	378 (84.4%)	1317 (81.9%)	687 (57.0%)
Negative	51 (22.9%)	70 (15.6%)	292 (18.1%)	518 (43.0%)
PR				
Positive	155 (69.2%)	359 (80.1%)	1116 (69.4%)	527 (43.7%)
Negative	69 (30.8%)	89 (19.9%)	493 (30.6%)	678 (56.3%)
HER-2				
Positive	62 (27.7%)	168 (37.5%)	352 (21.9%)	360 (29.9%)
Negative	162 (72.3%)	280 (62.5%)	1257 (78.1%)	845 (70.1%)
Molecular subtype				
HR+HER2−	133 (59.4%)	259 (57.8%)	1083 (67.3%)	527 (43.7%)
HR+HER2+	41 (18.3%)	128 (28.6%)	249 (15.5%)	189 (15.7%)
HR−HER2−	29 (12.9%)	21 (4.69%)	174 (10.8%)	318 (26.4%)
HR−HER2+	21 (9.38%)	40 (8.93%)	103 (6.40%)	171 (14.2%)
Visceral metastasis				
Yes	65 (29.0%)	8 (1.79%)	401 (24.9%)	702 (58.3%)
No	159 (71.0%)	440 (98.2%)	1208 (75.1%)	503 (41.7%)

**Table 2:** Univariate and multivariate analysis of the characteristics in the training cohort. OR: odds ratio; CI: confidence interval.

Characteristics	Training Cohort		Univariate Analysis		Multivariate Analysis	
	Bone metastasis (+)	Bone metastasis (−)	χ^2^	*p*	OR (95%CI)	*p*
Cases	*n* = 224	*n* = 448				
Age			0.361	0.604		
≤50	111 (49.6%)	211 (47.1%)				
>50	113 (50.4%)	237 (52.9%)				
Menopausal state			3.577	0.113		
yes	108 (48.4%)	240 (53.6%)				
No	115 (51.6%)	208 (46.4%)				
Histological grade			6.048	0.014	0.516 (0.337–0.790)	0.002
I & II	164 (73.2%)	284 (63.4%)				
III	60 (26.8%)	164 (36.6%)				
Affected side			0.168	0.681		
left	118 (52.7%)	245 (54.7%)				
right	106 (47.3%)	203 (45.3%)				
Maximum tumor diameter			2.373	0.305		
≤2 cm	79 (35.3%)	184 (41.1%)				
>2 cm ≤ 5 cm	138 (61.6%)	254 (56.7%)				
>5 cm	7 (3.12%)	10 (2.23%)				
N stage			0.527	0.468		
Positive	155 (69.2%)	296 (66.1%)				
Negative	69 (30.8%)	152 (33.9%)				
TNM-stage			<0.001	1		
I	36 (16.1%)	72 (16.1%)				
II	112 (50.0%)	224 (50.0%)				
III	76 (33.9%)	152 (33.9%)				
ER			4.689	0.03	1.120 (0.541–2.319)	0.760
Positive	172 (77.1%)	378 (84.4%)				
Negative	51 (22.9%)	70 (15.6%)				
PR			9.335	0.002	0.476 (0.308–0.736)	0.001
Positive	155 (69.2%)	359 (80.1%)				
Negative	69 (30.8%)	89 (19.9%)				
HER-2			5.97	0.015	0.502 (0.327–0.771)	0.002
Positive	62 (27.7%)	168 (37.5%)				
Negative	162 (72.3%)	280 (62.5%)				
Molecular subtype			20.046	<0.001		
HR+HER2−	133 (59.4%)	259 (57.8%)				
HR+HER2+	41 (18.3%)	128 (28.6%)				
HR−HER2−	29 (12.9%)	21 (4.69%)				
HR−HER2+	21 (9.38%)	40 (8.93%)				
Vessel carcinoma embolus			0.738	0.39		
Visible	63 (28.1%)	142 (31.7%)				
Invisible	161 (71.9%)	306 (68.3%)				
KI67			4.416	0.036		
≤30%	95 (42.4%)	230 (51.3%)				
>30%	129 (57.6%)	218 (48.7%)				
Nerve invasion			0.002	0.962		
Yes	21 (9.38%)	40 (8.93%)				
No	203 (90.6%)	408 (91.1%)				
Visceral metastasis			114.369	<0.001	26.831 (12.230, 58.864)	<0.001
Yes	65 (29.0%)	8 (1.79%)				
No	159 (71.0%)	440 (98.2%)				

## Data Availability

The data presented in this study are available upon request from the corresponding author due to ethical restrictions.
